# Performance Evaluation of NIPT in Detection of Chromosomal Copy Number Variants Using Low-Coverage Whole-Genome Sequencing of Plasma DNA

**DOI:** 10.1371/journal.pone.0159233

**Published:** 2016-07-14

**Authors:** Hongtai Liu, Ya Gao, Zhiyang Hu, Linhua Lin, Xuyang Yin, Jun Wang, Dayang Chen, Fang Chen, Hui Jiang, Jinghui Ren, Wei Wang

**Affiliations:** 1 BGI Education Center, University of Chinese Academy of Sciences, Shenzhen, 518083, China; 2 BGI-Shenzhen, Shenzhen, 518083, China; 3 Shenzhen People’s Hospital, the Second Clinical Medical School of Jinan University, Shenzhen, 518020, China; 4 Section of Molecular Disease Biology, Department of Veterinary Disease Biology, Faculty of Health and Medical Sciences, University of Copenhagen, 2200, Copenhagen N, Denmark; Tor Vergata University of Rome, ITALY

## Abstract

**Objectives:**

The aim of this study was to assess the performance of noninvasively prenatal testing (NIPT) for fetal copy number variants (CNVs) in clinical samples, using a whole-genome sequencing method.

**Method:**

A total of 919 archived maternal plasma samples with karyotyping/microarray results, including 33 CNVs samples and 886 normal samples from September 1, 2011 to May 31, 2013, were enrolled in this study. The samples were randomly rearranged and blindly sequenced by low-coverage (about 7M reads) whole-genome sequencing of plasma DNA. Fetal CNVs were detected by Fetal Copy-number Analysis through Maternal Plasma Sequencing (FCAPS) to compare to the karyotyping/microarray results. Sensitivity, specificity and were evaluated.

**Results:**

33 samples with deletions/duplications ranging from 1 to 129 Mb were detected with the consistent CNV size and location to karyotyping/microarray results in the study. Ten false positive results and two false negative results were obtained. The sensitivity and specificity of detection deletions/duplications were 84.21% and 98.42%, respectively.

**Conclusion:**

Whole-genome sequencing-based NIPT has high performance in detecting genome-wide CNVs, in particular >10Mb CNVs using the current FCAPS algorithm. It is possible to implement the current method in NIPT to prenatally screening for fetal CNVs.

## Introduction

Owing to the discovery of fetal cell-free DNA (cfDNA) in maternal plasma and rapid development of next-generation sequencing (NGS), noninvasive prenatal testing (NIPT) has brought confound changes to antenatal healthcare in the past few years[1]. The clinical validity and utility of NIPT for testing common aneuploidies have been endorsed by various clinical guidelines for using in high risk pregnancies[2]. Future application of NIPT may expand to average–risk pregnancies[2, 3]. However, chromosomal CNVs such as deletion and duplication remain a challenge for NIPT because of their small region of chromosomal abnormality[4]. CNVs are known to commonly exist in human genome, and diseases associated with CNVs, such as DiGeorge syndrome (22q11), Cri-du-chat syndrome (5p-), 1p36 deletion syndrome, are documented[5]. Postnatally, pathogenic CNVs are important contributors of intellectual disabilities in newborns, while in prenatal practice increasing evidence showed that disease-causative CNVs are associated with adverse pregnant outcomes. For instance, in samples with normal karyotype, clinically relevant CNVs were identified in 6% with ultrasound abnormalities and in 1.7% with advanced maternal age or positive serum screening results[6]. Recently, Dong et al showed that among samples referred to chromosomal analysis, 6.4% of samples of products of conception (e.g. spontaneous abortions and stillbirth), 13.5% of prenatal samples, and 26.3% of postnatal samples contained pathogenic CNVs[7]. Unlike aneuploidy, the risk of CNVs in fetus is independent of maternal age, and thus younger pregnant women may equally suffer the risk of pathogenic CNVs as older women[8]. Thus prenatal testing for clinically significant CNVs may bring benefit to clinical management and genetic counseling of pregnant outcome. Currently, amniocentesis or chorionic villus sampling (CVS) followed by karyotyping or microarray is the major approach to identify fetal CNVs, although a small but significant risk of miscarriage is associated with the procedures[9].

Several studies have showed the possibility of using whole genome sequencing-based NIPT to detect fetal CNVs [10–12]. However, these methods require very deep sequencing which significantly increases the cost and difficulty for clinical use. Recently, several proof-of-concept studies also evaluated the low-coverage sequencing method for the detection of fetal CNVs. For instance, Yin et al developed a method to identify 71.8% of CNVs using 3.5 million reads, but the performance dropped to 41.2% when CNVs were below 5Mb[13]. Straver et al reported the detection of large CNVs (over 20Mb) with low sequencing depth (0.15–1.66X) which had limited clinical value[14]. Lo et al, reported 64.5% (20/31) of accuracy when 4–6 million reads were used to analyze samples with 3Mb to 42Mb CNVs[15]. However, if CNVs were smaller than 6Mb, only 5 in 13 cases were identified. Previously we also reported a low-coverage sequencing method for CNV detection (referred as FCAPS)[16]. Using less than 8 million reads, the method can theoretically detect over 90% of >10Mb CNV at 10% fetal fraction. Although the accuracy appeared to be high, the FCAPS method was only confirmed with four clinical samples containing CNVs. Thus this method needs to be further validated with larger sample size. In this study, we evaluated the performance of FCAPS in detecting CNVs using selected clinical samples with known CNVs and calculate the estimated sensitivity and specificity.

## Methods

### Clinical samples

Since September 2011, maternal blood samples were obtained for NIPT service at BGI-Shenzhen with the following requirement: 1) maternal age was above 18 years; 2) gestational age was above 12 weeks; 3) singleton pregnancy. For NIPT test, 5ml of maternal blood from each woman was collected into an EDTA-containing tube with informed consent, and plasma was extracted for NIPT as previously described[17]. Exceeding plasma samples were dispensed and stored at -80°C. In this study, plasma samples that were confirmed by karyotyping or microarray as euploid or CNV-containing were retrospectively selected from our stored plasma collection from Sep 2011 to May 2013. The FCAPS was introduced into NIPT analytic pipeline after May 2013, thus the selected samples had not been pre-screened for CNV. This study was approved by the Institutional Review Board on Bioethics and Biosafety of BGI (BGI-IRB). Written informed consent was provided in this study, research did not be carried out until participants signed the agreement.

### DNA preparation and sequencing

QIAamp Circulating Nucleic Acid Kit (QIAGEN) was used to extract plasma cfDNA following the manufacturer’s instruction. Then cfDNA was prepared for library construction, quality control, and multiplexing for sequencing as described before[17]. Sixteen libraries were pooled and sequenced with 36-cycles sequencing using Illumina HiSeq2000 platforms. A barcode tracking system was employed during sample preparation. Sequencing reads were trimmed and aligned to a universal unique read set incised from the human reference genome (hg19, NCBI build 37). Risk of chromosomal aneuploidy was calculated using the binary hypothesis t-test and logarithmic likelihood ratio L-score as previously reported[17]. The FCAPS algorithm was used for CNV identification, which employed a regression-based GC correction strategy, binary segmentation for breakpoint localization, and dynamic threshold for signal filtering [16].

### Fetal fraction estimation by URY

Fetal fraction was calculated in male pregnancies using method described before[18]. Briefly, formula: $\varepsilon_{i,Y}=\frac{{\text{cr}}_{i,Y}-{\text{cr}’}_{i,Y,f}}{{\text{cr}‘}_{i,Y,m}-{\text{cr}‘}_{i,Y,f}}$ was used to calculate the fetal fraction estimate by chromosome Y of sample I (ε _*i*,*Y*_), in which cr′_*i*,j,m_ = *f*_*j*,*m*_(*GC*_*i*,*j*_)(*j* = *X*,*Y*) indicates the fitted relative k-mer coverage from a regression of an adult male data set, and cr′_*i*,*j*,*f*_ = *f*_*j*,*f*_(*GC*_*i*,*j*_)(*j* = *X*,*Y*) indicates the fitted relative k-mer coverage from a regression of a fetal female dataset.

### Evaluating performance of CNV identification

Before testing for CNV, identity information of the selected samples was removed. Samples were randomly re-arranged and blinded tested by laboratory and bioinformatics personnel. Testing results were compared to karyotyping or microarray results to calculate sensitivity and specificity. Clinical information such as ultrasound, amniotic fluid and maternal white blood cell detection could be also taken into account to support analysis of test results.

## Results

From September 1, 2011 to May 31, 2013 there were 919 samples with karyotyping or microarray results, including 21 samples with CNVs >10Mb, 7 samples with CNVs <10Mb, 5 samples with two CNVs (collectively referred as the positive sample set), and 886 euploid samples (referred as the reference set). Table 1 shows the demographic characteristics of the positive sample set, which contained CNV from 1Mb to 129Mb, including seven deletions causing Cri-du-chat syndrome (J01350, mic0014, mic0012, mic0005, HYQ19, H11001, H05010) and five reciprocal CNV syndromes (mic0009,Q00084, H34058, mic0017,H34056) (Table 2). Mean maternal age of the overall group was 31.71 years old, with the range of 20 to 38 years. Mean gestational age was 20.24 weeks, ranging from 12 to 37 weeks (Table 1). Maternal age of the positive sample set was compared to that of the reference set by T-test, and the p-value of 0.78 indicated no significant difference of the two groups. After removing sample identities, 919 samples were blinded sequenced and analyzed by FCAPS. No testing failure was reported. With 24 plex sequencing, each sample received on about 7M unique reads. Based on URY, fetal fraction of male pregnancies were 9.7% on average.

**Table 1:** Basic characteristic of the samples in this study.

**Total samples (n = 919)**	
Gestational weeks (Min—Max, Mean±SD)	12–37, 20.24±2.43
Maternal age (Min—Max, Mean±SD)	20–38,31.71±5.35
**Samples with CNVs (n = 33)**	
Gestational weeks (Min—Max, Mean±SD)	12–27, 18.24±5.50
Maternal age (Min—Max, Mean±SD)	22–38, 32.44±4.39
**Euploid samples (n = 886)**	
Gestational weeks (Min—Max, Mean±SD)	15–37, 20.31±2.20
Maternal age (Min—Max, Mean±SD)	20–38, 31.71±5.36

**Table 2:** Detailed information of 33 samples with known CNVs.

Sample ID	Fetal gender	Gestation (Weeks+Days)	Karyotype /array results		FCAPS result CNV location(kb)	Unique reads (M)	Fetal fraction by ChrY	Location accuracy	Pathogenicity ^b^
			CNV size(Mb)^a^	Banding					
Size of CNVs > 10Mb									
mic0009	Female	23+2	28	46,XX,dup(1)(p36)	dup(1)(555–4,882)	5.8	NA	Covered by karyotyping	Reciprocal to 1p36 microdeletion syndrome
218	Female	25+1	24.8	46,XX,del(4)(q24q26.1)	del(4)(131,274–136,761)	6.8	NA	Covered by karyotyping	NA
J01350	Female	19+0	15	46,XX,del(5)(p15.33p15.2)	del(5)(569–9,545)	8.1	NA	Covered by karyotyping	Cri du Chat Syndrome
mic0014	Female	17+4	13.6	46,XX,del(5)(p13)	del(5)(63–17,216)	6.9	NA	No overlap	Cri du Chat Syndrome
mic0012	Male	12+6	10.5	46,XY,del(5)(p14)	del(5)(608–20,832)	6.6	5.20%	Overlap 2.4Mb (<50%)	Cri du Chat Syndrome
mic0005	Female	13+2	25.6	46,XX,del(5)(p15.33p14.1)	del(5)(2,113–20,995)	6.9	NA	Overlap 18Mb (>50%)	Cri du Chat Syndrome
HYQ19	Female	20+4	48.4	46,XX,del(5)(p15p11)	del(5)(63–20,846)	5.5	NA	Covered by karyotyping	Cri du Chat Syndrome
H11001	Female	17+6	18.8	46,XX,del(5)(p15.32p14.3)	del(5)(4,884–21,072)	5.4	NA	Covered by karyotyping	Cri du Chat Syndrome
H05010	Male	18+1	18.4	46,XY,del(5)(p15)	del(5)(601–14,449)	7.7	14.30%	Covered by karyotyping	Cri du Chat Syndrome
K003762	Female	22+6	27.3	46,XX,del(9)(q22q32)	Undetected	6.5	NA	Undetected	NA
K000219	Female	22+4	11.8	46, XX, del(10)(q22.3q23.2)	Undetected	7.6	NA	Undetected	NA
661017	Male	13+3	22.5	46,XY,del(11)(q13.3q14.3)	del(11)(69,917–91,647)	5.6	4.70%	Covered by karyotyping	NA
D014LXY	Male	14+4	15.4	46,XY,del(18) (p11.3p11.2)	del(18)(913–8,064)	7.8	6.70%	Covered by karyotyping	NA
EF00011	Female	24+2	17.2	46,XX,del(18)(p11)	del(18)(483–14,400)	8.4	NA	Covered by karyotyping	NA
BR00166	Female	31+0	18.1	46,XX,del(18)(q21)	del(18)(38,987–53,333)	6	NA	Overlap 10Mb (>50%)	NA
L00626	Female	13+4	17.2	47,XX,mos +i(18)(p11)[7]/46,xx[23]	dup(18)(483–14,400)	8.5	NA	Covered by karyotyping	NA
DG00031	Male	17+0	24.2	46,XY,del(18)(q21.3q23)	del(18)(60,451–76,112)	5.2	17.10%	Covered by karyotyping	NA
J00051	Female	15+1	15.4	46,XX,del(18)(p11.32p11.21)	del(18)(483–14,400)	7.1	NA	Covered by karyotyping	NA
R03458	Female	20+0	20	46,XX,del(X)(q23q25)	del(X)(111,446–122,549)	8	NA	Covered by karyotyping	NA
146779	Female	19+2	60	46,XX,Xp-	del(X)(2,709–32,247)	6.1	NA	Covered by karyotyping	Steroid sulphatase deficiency (STS)
Q00084	Female	25+1	38.7	46,XX,del(X)(q24q28)	del(X)(96,252–148,245)	5.4	NA	Covered by karyotyping	Reciprocal to Pelizaeus-Merzbacher disease and Xq28 (MECP2) duplication
Size of CNVs < 10Mb									
BO00074	Female	28+1	5.5	46,XX,?del(7)(q32)	del(7)(140,295–158,820)	8.4	NA	No overlap	NA
LMQ155	Female	24+1	1.3	arr 13q21.2(60,399–61,730)×3	dup(13)(58,259–63,190)	7.6	NA	Overlap 1.3Mb (>50%)	NA
EH00601	Male	16+4	3	arr 13q31.1(80,281–83,294)×1	del(13)(81,667–85,377)	6	10.20%	Overlap 1.6M (>50%)	NA
H34058	Male	16+6	9.1	46,XY,del(15)(q26.2q26.3)	del(15)(96,390–102,497)	7.2	9.20%	Covered by karyotyping	Reciprocal to 15q26dupovergrowth syndrome
mic0017	Female	26+3	9	46,XX,del(15)(q26.2q26.3)	del(15)(93,346–102,429)	7.5	NA	Covered by karyotyping	Reciprocal to 15q26dup overgrowth syndrome
AL00944	Male	16+1	3.5	arr 18q11.2.q12.1(25,341–28,865) ×1	del(18)(23,066–27,505)	6.2	12.90%	Overlap 2.2Mb (>50%)	NA
ZNY162	Male	19+4	8.5	arr 18q22.3.q23(69,461–78,014)×1	del(18)(59,800–75,091)	7.4	16.20%	Overlap 5.6Mb (>50%)	NA
With two CNVs									
R02423	Female	23+1	Chr3:129.2 Chr14:3.7	46,XX,-14,+der(3;14)(q21;p13)	dup(3)(113,535–173,610), undetected	6.4	NA	Covered by karyotyping;undetected	NA
01HK67	Female	23+2	Chr4:13.6 Chr7:20.9	46,XX,del(4)(q34.3q35.2), dup(7)(p22p21.1)	del(4)(180,071–191,250), dup(7)(612–17,257)	5.4	NA	Covered by karyotyping;No overlap	NA
AR00208	Female	24+5	Chr5:58.2 Chr13:59.6	46,XX,der(5;13)(q15;q21),+13	undetectedT13, del(5)(569–13,119)	7.3	NA	Undetected;Covered by karyotyping	Cri du Chat Syndrome
H34056	Male	21+5	Chr12:12.1 Chr17:3.5	46,XY,dup(12)(p13.33p13.1) del(17)(q25.3)	dup(12)(604–12,741),del(17)(77,596–81,055)	7.3	17.60%	Covered by karyotyping;Overlap 3.5Mb (>50%)	Reciprocal to 12p13.33 Microdeletion Syndrome;NA
mic0016	Male	18+4	Chr13:3.7Chr6:1	46,XY,del(13)(q31.1),del(6)	undetected	6	10.20%	undetected	NA

^a^ size calculated as the upper limit based on karyotyping data

^b^ data from decipher database (http://decipher.sanger.ac.uk)

**Fig 1:**
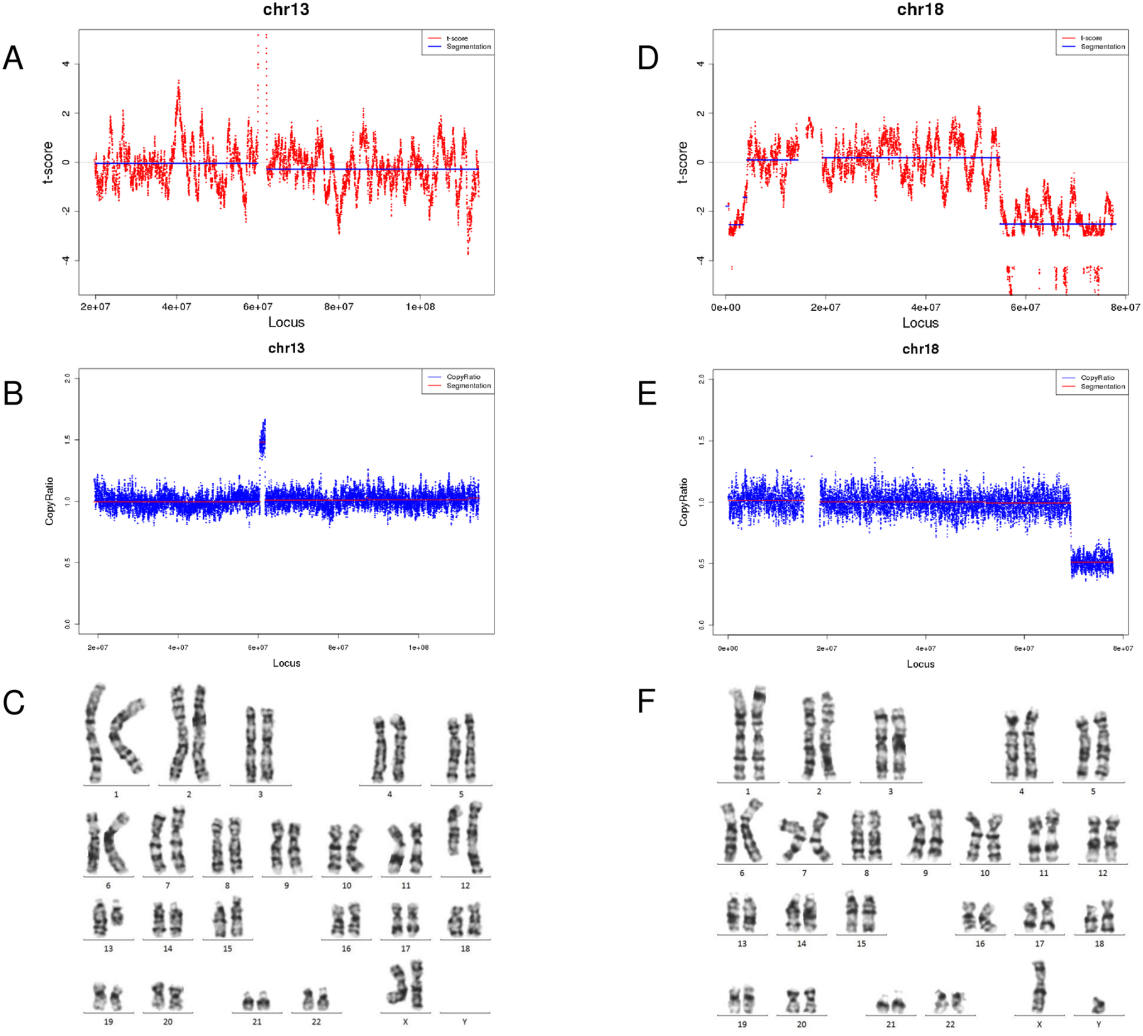
Examples of FCAPS results and amniotic fluid confirmation of two cases (LMQ155 and ZNY162) with CNVs<10Mb. A-C, (A) FCAPS result, (B) amniotic fluid sequencing result and (C) karyotyping result of sample LMQ155, which contained a 1.3Mb microduplication on chromosome 13; D-F, FCAPS result (D), amniotic fluid sequencing result (E), and karyotyping result (F) of ZNY162, which contained a 8.7Mb deletion on chromosome 10.

In the positive sample set, at such low sequencing depth, CNVs were detected in 33 samples by FCAPS (Fig 1). When stratified by CNV size, FCAPS identified 25 samples containing 27 events of CNVs> 10Mb, and 10 samples containing 11 events of CNVs<10Mb (Table 2). Three samples with CNV>10Mb (K003762, K000219, AR00208) and two samples with CNV<10Mb (R02423, mic0016) were undetected by FCAPS. In samples containing multiple CNVs, the same number of CNVs as karyotyping or microarray was identified by FCAPS in 01HK67 and H34056, while in R02423, AR00208, mic0016 CNV was partly identified. In twenty four samples, the CNV locations identified by FCAPS were fully covered or at least 50% overlapped comparing to the karyotyping/microarray results, which were classified as ‘Consistent’ (Tables 2 and 3). In contrast, in five samples FCAPS predicted the CNV on correction chromosome yet with small (<50%) or no overlap to karyotyping/microarray results, thus were classified as ‘Partly Consistent’.

**Table 3:** Performance of detecting CNVs events in 919 pregnant women who took karyotyping/microarray testing.

Size of CNV	Consistent^a^	Partly consistent^b^	Inconsistent^c^	Sensitivity	Specificity
>10Mb	21	3	3	88.89%	99.32%
<10Mb	7	1	3	72.73%	99.09%
Total CNVs	28	4	6	84.21%	98.42%
Euploid	872	0	14		

^a^. CNVs with FCAPS locations covered or >50% overlapped by karyotyping/microarray locations were classified as consistent

^b^. CNVs with FCAPS locations <50% or no overlapped by karyotyping/microarray locations were classified as partly consistent

^c^. CNVs with FCAPS results that could not be confirmed by karyotyping/microarray results were classified as inconsistent

**Fig 2:**
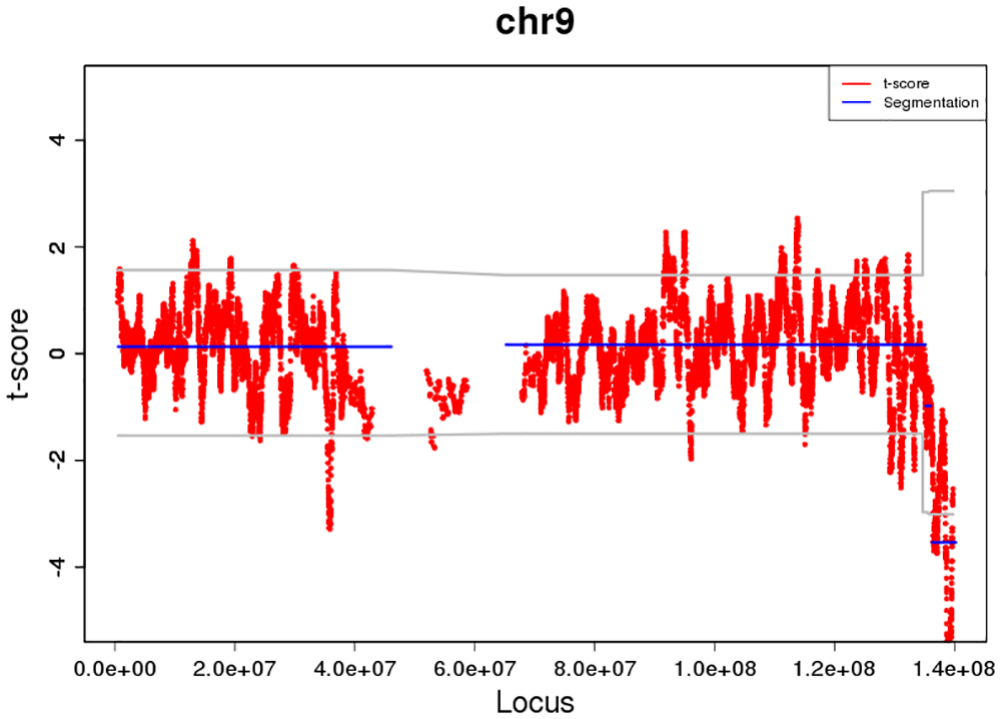
False positive results of sample INC6 by FCAPS showing CNV location near to telomere.

In total of 886 euploid samples, 872 had negative results from FCAPS analysis, resulting in 14 false positive results including 6 CNVs>10Mb and 8 CNVs<10Mb (Table 4). In these 14 false positive samples, 4.3–7.7Mb unique sequencing reads were obtained in each sample and the fetal DNA fraction tested in male pregnancy were 5.4–10.3%, showing consistent sequencing depth to the previous report [16]. Four in these fifteen false positive results were caused by maternal CNV backgrounds, as showed by sequencing maternal white blood cells (Table 4). One false positive case (INC6) had CNV signals close to chromosome telomere (Fig 2). CNVs of the other six false positive cases were less than 10Mb.

**Table 4:** Detailed information of samples with inconsistent NIPT-FCAPS results to karyotyping/microarray results.

Sample ID	Fetal gender	Karyotyping/microarray result	FCAPS resultsCNV Location (kb)	Other clinical information	Unique Reads (M)	Fetal fraction by ChrY
hc00001	Female	46,XX	del(2) (10,351–25,653)	WBC: arr[18]2p25.1p23.3(10,284,910–25,332,072)x1	6.6	NA
R00033	Male	46,XY	del(4)(27,923–35,991)	Normal ultrasound result	5.2	7.70%
Y00204	Male	46,XY	del(5)(569–25,112)	NA	4.6	5.40%
C00473	Male	46,XY	del(6)(114,064–115,869)	Normal FISH and ultrasound result	6.4	7.00%
X00115	Female	46,XX	dup(8)(35,321–42,958)	Normal ultrasound result	5.7	NA
INC6	Female	46,XX	del(9)(122,500–134,200)	NA	4.3	NA
R01244	Female	46,XX	dup(13)(19,351–24,028)	Normal ultrasound result	4.3	NA
L00120	Male	46,XY	dup(13)(22,127–26,733)	Normal ultrasound result	5.3	10.30%
A00085	Female	46,XX	del(13) (46,586–57,489)	WBC: del(13)(q21.1, 14M)	5.7	NA
T00002	Male	46,XY	del(13) (51,037–56,569)	WBC: del(13)(q14.3-q21.1, 4.89M)	6.1	6.50%
B00560	Male	46,XY	dup(13)(108,324–111,151)	WBC: dup(13)(q34)Normal ultrasound result	7.5	8.10%
F01220	Female	46,XX	dup(15)(73,624–98,091)	Normal ultrasound result	7.3	NA
N00071	Male	46,XY	dup(20)(454–24,767)	Normal ultrasound result	6.5	7.10%
W00930	Male	46,XY	dup(21)(22,756–25,425)	Normal ultrasound result	7.7	8.40%

WBC, white blood cell; NA, not available.

To calculate the performance of FCAPS, CNV events that were classified as consistent and partly consistent were treated as true positive results, and CNVs classified as inconsistent were used as false positive or false negative results (Table 3). For CNV>10Mb, sensitivity and specificity were calculated as 88.89% (95%CI: 70.84%-97.65%) and 99.32% (95%CI: 98.52%-99.75%) respectively. For CNV<10Mb, sensitivity and specificity were 72.73% (95%CI: 39.03%-93.98%) and 99.09% (95%:98.22%-99.61%) respectively. Collectively, FCAPS produced 84.21% (95%CI: 68.75%-93.98%) of sensitivity and 98.42% (95%CI: 97.36%-99.13%) of specificity in detecting CNVs.

## Discussion

Using NIPT for CNV detection was showed to be possible[19]. However, NIPT efficacy of CNV detection has not been extensively evaluated, mainly due to the lower disease prevalence [20]. Previously we developed a method to noninvasively detect CNV, which relied on GC-bias correction, binary segmentation, and dynamic threshold for signal filtering to reduce sequence variability and improve accuracy [16, 21]. In this study, we evaluated the efficacy of CNV detection using archived samples and showed that CNVs>10Mb can be detected with high sensitivity whereas CNVs<10Mb have reduced detection rate.

In the selected samples with CNVs ranging from 1 to 129Mb, the FCAPS method showed the total sensitivity of 84.21% and specificity of 98.42%. Our method showed relatively high efficacy in detecting CNVs bigger than 10Mb, and the efficacy reduced when testing in CNVs smaller than 10Mb. This trend fits our previous *in silicon* simulation, as well as other studies showing that reduced CNV size leads to decreased detection power[16]. In general, our method generated 46 positive CNV results in which 32 were consistent to karyotyping/microarray confirmation, leading to a 69.57% of accuracy. However, the real positive predictive value of our method could be different in practice, since the CNVs samples were from a selected group and the occurrence rate did not represent that of a normal pregnancy population. Several previous studies reported their preliminary results of the performance of noninvasive CNV detection. However, it is difficult to compare their results with ours because different sequencing platforms[13], sequencing parameters[13, 15], and CNV sizes[14] were involved. Nonetheless, factors affecting performance of CNV detection were commonly suggested by these studies, including CNV size, sequencing depth, fetal fraction, and GC contribution. In this study, the selected samples included a wide range of CNV sizes on different chromosomes, as well as various types of CNVs such as reported microdeletion or microduplication syndromes, imbalanced translocations, and CNV mosaicism. The ability of identifying CNVs of different size and types with relatively high accuracy implies that whole-genome sequencing-based method benefits the identification of genome-wide CNVs without prior knowledge of their locations.

Maternal CNV background has been reported to induce NIPT false positive results[13, 17]. This is in consistent to our data that maternal white blood cells were available for verification in four false positive cases, all confirmed with maternal CNV backgrounds. Another sample had a CNV close to telomere. Due to the lack of maternal white blood cells, the false positive reason could not be validated. However, telomere sequence may be prone to have false positive or false negative results[22]. Among the remaining nine false positive samples, six had CNVs at submicroscopic level, which may be difficult to confirm by karyotyping method due to limited resolution[22]. Thus our data support the use of microarray for prenatal diagnosis owing to better resolution, as suggested by the American College of Medical Genetics and Genomics and the American College of Obstetricians and Gynecologists[23–25].

The existence of false positive results and the fact that current NIPT method cannot distinguish the source of CNV (maternal background or fetal origin) indicate that CNVs identified by NIPT should be confirmed by prenatal diagnosis and maternal background testing to provide the comprehensive information for post-test genetic counseling. However, this may significantly increase the screening cost and thus reduce the clinical utility of screening for CNV by NIPT[15]. Nonetheless, the decrease of NIPT cost and improvement of accuracy in the future may improve the cost-effectiveness of CNV screening and overcome this barrier for clinical use. Furthermore, the existence of false negative result of CNV detection in our study as well as previous other studies implies that a negative result of CNV screening by NIPT cannot rule out the possibility of clinically significant CNVs. Thus other clinical information such as ultrasound result should be also taken into account to interpret result and provide post-test counseling.

Several limitations remained in this study. Firstly, fetal fraction and confirmation of maternal CNV background was only available in limited samples, which impedes the difficulty in explaining false positive and false negative results. Secondly, due to the low occurrence rate of CNV in prenatal samples, limited number of samples was selected from archived storage, thus the clinical performance of our method in particular the positive predictive value could not be assessed. Moreover, the positive samples may not well-represent clinically significant CNVs which are commonly less than 3.5 Mb[26]. Further studies using ideally prospective CNV samples are needed for clinical validation of the method.

## Conclusion

In conclusion, our study showed a high sensitivity and specificity in detecting CNVs over 10Mb using a low coverage sequencing method, which is consistent to the previous *in silicon* analysis. The method also appeared to have good performance in detecting CNVs smaller than 10Mb but further evaluation is still required. Our results demonstrated that noninvasive prenatal screening for fetal CNVs is promising for clinical use although its clinical utility needs to be further studied.
